# Unseen Obstacles: Gender Leadership Disparities in Public Health Academia

**DOI:** 10.3389/phrs.2024.1608122

**Published:** 2024-12-09

**Authors:** Mohamed Osman Gafar Abdalla, Aleksander Sobczyk, Geri Kemper Seeley

**Affiliations:** World Federation of Public Health Associations (WFPHA), Geneva, Switzerland

**Keywords:** gender disparity in leadership, public health academia, leaky pipeline effect, higher education policy, inclusive policy-making

The World Federation of Public Health Associations (WFPHA) Global Public Health Education Benchmarking Project (GPHEBP) is designed to assess public health programs worldwide, fostering alignment among existing programs, schools, and institutions. Higher education globally faces significant challenges, such as addressing resource disparities, adapting to technological advancements, and fulfilling the dire need for interdisciplinary approaches [1, 2]. These challenges are intensified in the field of public health by the urgent need to ensure that teaching staff are experienced, competent, and capable of tackling global health threats such as pandemics, health inequities, and increasing man-made and natural disasters [3]. The WFPHA, a worldwide professional society of public health associations, addresses these issues through 11 working groups including the Public Health Education and Training Working Group (PETWG), which focuses on improving educational frameworks and ensuring that public health training equips future professionals to meet evolving global health challenges.

A paramount focus of the GPHEBP is the assessment of gender disparity in public health academia leadership to determine whether leaders are truly representative of the broader public health workforce. The project initially faced challenges in surveying gender comprehensively due to differing social norms across regions, requiring the study to specifically center on sex, defined as the biological characteristics that define humans as female or male [4]. A central hypothesis of the GPHEBP is that public health academia leadership should ideally mirror the global sex ratio—approximately 1:1—where men and women share leadership positions equally. Despite women representing around 70% of the global healthcare workforce (including public health) as a whole, women remain substantially underrepresented in leadership roles [5]. This disparity hinders the ability of public health systems to benefit from diverse leadership, which is essential for comprehensive health policy development and decision-making.

The GPHEBP project is designed to retrieve data from websites of accredited public health institutions measuring on a range of indicators including the man-to-woman ratio within leadership roles, particularly among deans. The diverse composition of the research team, including members from various academic, and professional backgrounds, hailing from different regions, further enriched the analysis. Project team members’ diversity enabled the disparities to be discussed and approached with sensitivity to regional and cultural nuances, ensuring that the results reflect an authentically global understanding of sex identification using these parameters.

Findings reveal noticeable gender disparities in leadership, with **111 males (45%)** and **94 females (38%)** in public health leadership positions, while **13% of the leaders’ sex remained unknown** due to a lack of publicly available visual or self-identifying information. These figures demonstrate the disproportionate representation of men in leadership, despite women’s notable presence in the public health workforce.

Furthermore, the results show that while the Americas region (AMRO) has made progress toward gender parity in public health leadership, WHO regions like Africa (AFRO) and the Middle East (EMRO) still face significant challenges. However, since most of the data came from US and Europe-based institutions, the accuracy of region-specific conclusions is limited. This highlights the need for more inclusive research to better understand the barriers faced by women in leadership across diverse contexts.

## Academia’s Smaller Gender Disparity

While gender disparities in public health leadership are evident, the project highlights that **academia has a smaller gender disparity** in comparison to clinical or policy-making leadership. However, this still falls short of true parity. The **“leaky pipeline” phenomenon**, where women’s progression slows at senior career stages, remains a significant barrier [6]. Women face additional challenges in reaching top positions due to systemic biases, lack of mentorship, and fewer opportunities for promotion [7].

Academia has made progress in promoting women yet the pace of change remains slow. This is largely because many institutions still lack transparency in hiring and promotion processes, perpetuating the underrepresentation of women in senior roles [8].

## Implications of Gender Disparity

The lack of gender diversity in public health leadership undermines the effectiveness of health systems. Gender-diverse leadership has shown improved decision-making, particularly in areas affecting Sexual and Reproductive Health and Rights [9]. Balanced teams bring a wider range of perspectives to the table, which leads to more innovative solutions and better outcomes for communities.

The underrepresentation of women in leadership roles limits attention to inclusive health policy development on issues disproportionately affecting women, such as maternal health or gender-based violence. Failing to address gender disparities in leadership can reduce the productivity and innovation of public health organizations, which in turn affects the overall efficiency of health systems [10].

## Recommendations for Change

To tackle these disparities, the research team recommends several strategies informed by extensive research in public health leadership and gender disparities:

1. Mentorship Programs: Developing mentorship programs that specifically support women in advancing to leadership roles is crucial. Such programs can help bridge the gap by offering guidance and opportunities for career growth.
2. Transparent Recruitment and Promotion Pro**cesses**: Ensuring transparency in hiring and promotion processes is essential for overcoming the barriers women face. Clear criteria and accountability mechanisms should be established to promote gender equity.
3. Leadership Preparation: Increased investment in leadership training programs tailored to women, especially in regions where gender disparities are most observed, can help prepare future female leaders.
4. Context-Specific Solutions: Given the regional variations highlighted by the data, it is essential to design context-specific solutions that address the unique challenges women face in different parts of the world, especially in conflict-affected or resource-limited settings. This includes addressing cultural and societal norms that hinder women’s progress in leadership roles, especially through policy solutions.

While there has been some progress toward equity in public health leadership, the findings of the study reveal that **considerable disparities remain**. With females holding only **38%** of leadership roles and stark regional variations in representation, clearly more remedial work needs to be done. These disparities are shaped by various factors beyond gender alone, highlighting the need for an intersectional approach. Aligning public health leadership with the global sex ratio and promoting gender equity is not just a matter of fairness; it is crucial for enhancing the effectiveness and inclusive nature of global public health systems.

By implementing targeted interventions and fostering gender diversity in leadership, the public health sector can better respond to the complex health challenges of the 21st century. Gender equality is not a luxury that only wealthy nations should prioritize; it is a fundamental requirement for building equitable and resilient communities. Institutions must actively survey these disparities and develop practical solutions to bridge the gap within their contexts, advancing both fairness and effectiveness in public health leadership.
